# Effects of Amalgam Fillings upon the System

**Published:** 1886-04

**Authors:** E. A. Bogue

**Affiliations:** New York.


ARTICLE IV.

### EFFECTS OF AMALGAM FILLINGS UPON THE SYSTEM.

BY E. A. BOGUE, M. D., NEW YORK.

(Read before the Dental Society of the State of New York.)

The subject given me to speak upon I consider a most unfortunate one—"The Effects of Amalgam Fillings upon the System." There are none, so far as proven. But as at least a few words are expected from me, I take the liberty of bringing before you the results of a few experiments that have been made, and a little of the guess-work that has been published since 1882. I was unable to find any record of experiments made previous to that time upon the subject, although I have looked back for forty years. Since then, Dr. Hitchcock, Dr. Talbot and Dr. S. P. Cutler, formerly of New Orleans, have all made experiments, most of which have been carefully conducted. The conditions which existed in the mouth have been detailed, and I will briefly rehearse a few of the principal experiments and the conclusions drawn from them.

Dr. Talbot's paper is published in the November number of the *Ohio State Journal.* I may, perhaps, be pardoned for rather sharp criticisms, for the time is short, and I am fully aware that sharp criticisms will be applied to what I say.

Dr. Talbot says in his experience: "A sufficient portion of the material is rubbed in the hand and placed in the tooth." His article is exceedingly loose in that particular. Good amalgam fillings are not made in that way, but the constituent parts are carefully weighed and the proper proportions preserved. Eight experiments were made by Dr. Talbot, by heating amalgam fillings in bottles to about 100 degrees, and a vapor was given off, blackening test paper; yet this same article notices other large amalgam fillings which were kept three months at 100 degrees that showed no change, and in others he notes an increase in weight from oxydation that has taken place.

Dr. Talbot fails to realize the difference between amalgam placed and kept continually in a hot and dry position and amalgams kept most of the time wet by the saliva.

Amalgams well made will not part with their mercury under about 400 degrees.

He speaks of cases of poisoning caused by amalgam fillings, and giving the verdict of a coroner's jury would have you believe that death was caused by the "existence of the amalgam in a second molar tooth on the right side of the lower jaw." The loose talk of an average coroner's jury is given as a scientific fact.

My own experiments in 1883 explain some other facts. A large variety of amalgam fillings were kept by me at 100

degrees for three months, but were kept in saliva, and they seemed to gain in weight. They were all weighed down to one-tenthousandth of a gramme. But in all the varieties tested by me there was no mercury found in the bottle, either by myself or Prof. Chandler, who tested the specimens after me to prevent the possibility of error.

Dr. Pease, of Dayton, has made some experiments, it seems, and as he is an old practitioner and a man of decided good sense, judging from his writings, I have the pleasure of noting what he says in the *Ohio State Journal* for September, 1882.

Dr. Pease says he has never seen any bad effects in any of his patients from amalgam fillings used in connection with gold, and he then calls attention to the blue mass pill so often administered, which take three days to work off. He asks what becomes of the mercury lying in the system during those three days; has it or has it not been absorbed? He assumes that there are cases of administration of mercury from drugs where poisoning would occur more than from fillings in the teeth.

If amalgams are inserted with a large surplus of mercury, the metallic mercury may be found in the mouth after the pressure that is applied to the filling brings the mercury to the surface, and from there is carried to the stomach as metallic mercury. I have read within the past two months of something like two pounds being put into the stomach of a patient by means of a tube to correct an impaction of foul matter, and in a few days the mercury was discharged and the patient then recovered.

We might argue, I think, that metallic mercury was harmless unless converted into an oxide or chloride.

I once more refer to Dr. Pease, who is exceedingly apt. He says if mercury exudes from a tooth through a defective filling, don't blame the mercury, but the dental colleges who license such men to do dental work.—*Odontographic Journal.*

